# To See or Not to See: A Systematic Review of the Importance of Human Ocular Surface Cytokine Biosignatures in Ocular Allergy

**DOI:** 10.3390/cells8060620

**Published:** 2019-06-20

**Authors:** Esrin Aydin, Moneisha Gokhale, Serap Azizoglu, Cenk Suphioglu

**Affiliations:** 1 Deakin Optometry, School of Medicine, Deakin University, Geelong, Waurn Ponds, Victoria 3216, Australia; eaydi@deakin.edu.au (E.A.); moneisha.gokhale@deakin.edu.au (M.G.); serap.azizoglu@deakin.edu.au (S.A.); 2 School of Life and Environmental Sciences, Deakin University, Geelong, Waurn Ponds, Victoria 3216, Australia

**Keywords:** ocular allergy, allergy, keratoconus, cytokine, biosignature, biomarker, immunoblot, contact lens wear

## Abstract

Cytokines are key cell signalling proteins in a number of immune and homeostatic pathways of the human body. In particular, they mediate intracellular mechanisms of allergy on the ocular surface by triggering cellular responses that result in typical physiological ocular allergy symptoms, such as itchiness, watery eyes, irritation, and swelling. Given the recent research focus in optometry on the aetiology of corneal ectasia subtypes like keratoconus, there is an increasing need for the development of new clinical diagnostic methods. An increasing trend is evident among recent publications in cytokine studies, whereby the concentrations of cytokines in healthy and disease states are compared to derive a specific cytokine profile for that disease referred to as ‘biosignatures’. Biosignatures have diagnostic applications in ocular allergy as a cheap, non-invasive alternative to current techniques like IgE antibody testing and skin prick tests. Cytokine detection from tear samples collected via microcapillary flow can be analysed either by enzyme-linked immunosorbent assays (ELISA), multiplex magnetic bead assays, or immunoblot assays. Characterising patient hypersensitivities through diagnostic tests is the first step to managing exposure to triggers. Investigating cytokine biosignatures in ocular allergy and their links to physiology are imperative and will be the focus of this systematic review article.

## 1. Introduction

The global impact of allergy is on the rise, with over 40% of the population currently suffering from some level of hypersensitivity [1]. Allergy indiscriminately affects people of all ages, sexes, ethnicities, and backgrounds. Collecting data in a large group of highly varied participants and allergic phenotypes over different seasons of the year is beneficial for updating prevalence data. The last longitudinal global prevalence report was completed by International Study of Asthma and Allergies in Childhood in 2011, the primary goals of which were to establish allergy prevalence in children, aged 6–14 years over different seasons of the year. An updated version of the same scale longitudinal study would provide invaluable insight into the global burden of disease of allergy today [2]. Recent epidemiological studies in the United Kingdom predict that the incidence will continue to rise, with up to 50% of Europe projected to suffer from some form of allergy by 2025 [3]. In Australia alone, the total wholesale cost of drugs used to symptomatically treat hay fever totalled $226 million in 2010, more than double the $108 million spent in 1991 [4]. Given the diverse range of symptoms, from itchy eyes and runny noses to anaphylaxis and throat closure, there is an increasing demand for medical intervention in diagnostics and long-term allergy treatment.

Current diagnostic methods for allergy rely heavily on patient history, subjective physical assessment, and grading of ocular symptoms. Alternative objective tests are invasive, requiring blood samples or multiple pricks with small volumes of allergens in a skin prick test (SPT) [5]. Blood samples are clinically used to quantify IgE antibodies generated in response to triggers like pollen, mould, or airborne spores [6,7]. Total IgE levels are then used to estimate the level of hypersensitivity, after which patients are expected to avoid triggers and treat themselves symptomatically during a flare-up [8]. Unfortunately, serum IgE testing is expensive [9] and may be inaccurate [10], as IgE antibody levels for an allergen do not necessarily correlate to individual hypersensitivity, and may flag false positives [8]. IgE level is therefore used as an estimate, although there is a clinical gap in the development of an alternative objective, non-invasive diagnostic test. Recent advances in research suggest that cytokine levels in tear film collected non-invasively from pooled basal tears on the bottom of the eyelids are a promising option [9].

To diagnose ocular allergy conditions with tear cytokines, preliminary research into ocular allergy and regulatory tear cytokines is imperative. Ocular allergy is a localised allergy occurring in 40–60% of allergic populations [11]. Key features of ocular allergy include itchiness, watery eyes, inflammation, conjunctivitis, and swelling of the ocular surface and eyelids [11]. Attempting to relieve symptoms by rubbing the eyes exacerbates inflammation and itchiness by triggering mechanical stress responses [12]. Chemotactic cytokines in stress responses attract repair cells to the conjunctiva to reduce damage; however, there is only so much this mechanism can reverse [13]. The impact of continued stress on the eye could be detrimental [14], as downstream effects include corneal ectasia, sleep disturbances, blurred eyesight, and the reduced ability to function in professional and daily life settings [15]. To decrease the prevalence of corneal ectasia among ocular allergy sufferers, early diagnosis and patient education regarding eye-rubbing is important [12]. It is envisaged that measuring cytokines in tears may lead to more definitive diagnosis and severity grading of ocular allergy. This review will investigate significance of cytokines in tears to help drive further research on the various types of ocular allergy, treatments, and management options.

Ocular allergy is divided into four clinical subtypes: seasonal allergic conjunctivitis (SAC) and perennial allergic conjunctivitis (PAC) are most common, followed by vernal keratoconjunctivitis (VKC) and atopic keratoconjunctivitis (AKC) [16]. SAC and PAC are the main focus of this review, due to their significance in the global burden of disease of ocular allergy, with SAC and PAC causing 90% of ocular allergy cases in the United States as of 2012 [17]. Each subtype is diagnosed based on symptoms and duration as outlined in Table 1. PAC has a comparable physiology to SAC with primary differences in acute or chronic presentation. PAC is an easier subtype to assess as tear samples can be collected during an active year-round flare-up, and not just in peak allergy seasons. In terms of the cellular mechanisms behind SAC and PAC, there does not appear to be any difference in allergic pathways.

## 2. IgE-Mediated Mechanism of Ocular Allergy

There are a number of cellular changes that occur among ocular allergy patients during an episode [18,20]. Most of these changes are due to IgE-mediated inflammatory immune responses [18]. Normal allergic responses in the eye involve the production of IgE, specific chemotactic and immunologic cytokines, and the stimulation of mast cells [11]. In short, local antigen presenting cells (APCs) in the conjunctiva recognise and digest allergens into allergic peptides [21]. Due to the abundance of T helper 1 (Th1) and T helper 2 (Th2) type CD4+ T helper cells within the conjunctiva, allergic peptides bind to receptors on naive Th2 cells, activating them and thus triggering overexpression of chemotactic cytokines that attract a number of naive B cells to the area [21]. T cell subtypes other than CD4+ helper T cells do not appear to influence the ocular allergy pathway [22], which is Phase I activation of B cells [21]. Phase II occurs when the B cell is re-exposed to the allergic peptide and transforms into a mature plasma cell capable of producing IgE antibodies specific to the allergen [21]. Allergic peptides come into contact with the conjunctiva and bind to specific IgE antibodies expressed on the surface of local mast cells [23]. This binding triggers an immediate conjunctival allergic response whereby mast cells degranulate and release histamine and a number of other mediators that cause physiological symptoms like redness, itching, and inflammation [23]. Unfortunately, attempting to rub the eyes worsens the irritation and stimulates the release of mechanical stress response molecules that add to these symptoms [12]. Cytokines, therefore, most effectively mediate the ocular allergy pathway by interacting with the upregulation of IgE upon exposure to allergic peptides.

## 3. Overview of Cytokines in Ocular Allergy

**Figure 1:**
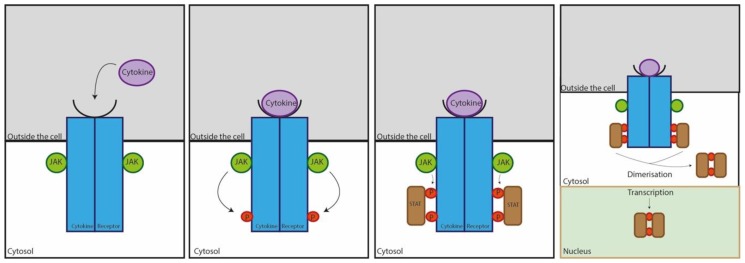
Janus kinase (JAK)/signal transducer and activators of transcription (STAT) pathway of cytokine action. Cytokines bind to receptors embedded in the cell surface, triggering the activation of one or more JAKs. The JAK then phosphorylates the receptors, and when the STATs bind, they are phosphorylated by receptors. Once the STAT is phosphorylated, it acts on the transcription and translation of DNA within the nucleus to regulate gene expression. The cell will then induce a signalling pathway, upregulate or downregulate more cytokines, or activate other cell types [26,27].

Physical symptoms of ocular allergy on the surface of the eye are controlled by interactions between cells and cytokines on a subclinical level [24]. Cytokines are communication polypeptides present in every cell of the body [24]. These polypeptides mediate most biological functions in the eye, controlling inflammation, chemotaxis, and activation of cells in immune and homeostatic pathways [25]. Their primary mode of action alters gene expression via Janus kinase (JAK)/signal transducer and activators of transcription (STAT) pathways, as outlined in Figure 1 [26,27]. Due to their versatile nature, each cytokine is capable of multiple functions dependent on their location in the eye [21]. In tear film, cytokines have roles in homeostasis, mechanical stress, and immune responses triggered by allergens [25].

## 4. Physiology of Ocular Allergy

This review focuses on deviant levels of regulatory cytokines in ocular allergy and their theoretical clinical applications in diagnosis and in the rationalisation of ocular allergy symptoms. A vast majority of cytokine concentration increases are linked to one another due to not-yet clearly defined biochemical mechanisms of ocular allergy. Table 2 below outlines what is known about cytokines in regard to inflammation and allergy. There are very few papers on ocular allergy-specific cytokine interactions, perhaps due to their highly multifaceted involvement in many biochemical pathways. This may be an interesting avenue for research.

What is known about cytokines in allergy, however, is that they play a major role in the upregulation of IgE and may thus have a direct link to the magnitude of allergic responses [28]. Increased concentration of cytokines like IL-4 and IL-13 are most commonly attributed to ocular allergy due to the promotion of IgE production when they act on Th2 cells [28]. Additionally, IL-4 and IL-13 play a role in mucus secretion and chemotactic cell recruitment to areas of inflammation. In the eye, these roles manifest as localised swelling and conjunctivitis [28]. Conversely, increased concentrations of IFNγ produced by Th1 cells have been shown to actively inhibit these cellular pathways by suppressing the activation of Th2 cells while increasing IL-12 production occurring via APCs [29]. In turn, IL-12 will then continue to upregulate IFNγ and thus suppress allergic reactions occurring on the ocular surface [29]. Other commonly occurring cytokines in allergy, like IL-1β, IL-2, IL-6, IL-8, IL-10, and TNFα, have largely undefined roles but are involved either in inflammation or the stimulation of cytokines with more major impacts on these biochemical pathways.

## 5. Tear Cytokine Analysis Methods

### 5.1. Immunoblotting

There are a number of techniques available for analysing cytokine levels in the tears of ocular allergy sufferers. In 2000, Schultz and Kunert used polyacrylamide gel electrophoresis (PAGE) followed by immunoblotting to determine the concentration of IL-6 in the tears of contact lens wearers [34]. This process, although inexpensive, is not time effective and is a highly involved method with ample opportunity for human error [35]. Additionally, human cytokine levels in tears occur in minute concentrations, and the immunoblot technique does not have sufficient sensitivity to accurately measure them [36]. Low level throughput and duration per run contribute to the low efficacy of this assay [35]. A more effective measure of cytokine concentration, such as ELISA, shows base levels of IL-6 in control tears that were unable to be detected by immunoblotting alone [34,37].

### 5.2. ELISA and Multiplex Assays

Multiple studies of inflammatory mediators in allergy rely on ELISA for quantitative analysis, due to the high sensitivity and reproducibility of results [38]. The primary downfalls of this method are the volume of sample needed per run, duration, and high cost [35]. Despite using non-invasive tear collection methods, like microcapillary tear suction, low volume samples are common, particularly in participants with dry eyes [38]. Tear collection by microcapillary may also agitate the lower lash line, triggering the formation of highly dilute reflex tears [39]. On the opposite end of the spectrum, older subjects are typically prone to dry-eye and, as such, may struggle to provide sufficient sample volumes. Dilution after collection helps, although it may impact on the accuracy of readings with low cytokine concentrations. A small sample size makes repeated measures impossible, calling the validity of the experiment into question as statistical analysis may not be possible [38]. Many researchers have attempted to side-step this without compromising accuracy by combining high-throughput magnetic bead microarrays with ELISA, with great success [40,41,42]. This approach has helped to reduce the number of runs and the time taken as cytokines are simultaneously measured for each sample [40]. Microassay plates also have the capacity to test more samples per plate than ELISA, making them cheaper overall [40].

## 6. Results of Previous Ocular Allergy Studies

### 6.1. Normal Tear Cytokine Levels

Given the important regulatory role of the tear film, most cytokines detected in healthy subjects are inflammatory. A summary of these regulators is shown in Table 3 below. Cytokines in the tear film drive the synthesis of other chemotactic and inflammatory cytokines during allergen invasion [25]. Since many cytokines exist at detectable levels in healthy human tears, studies on ocular allergy sufferers’ tears use control data to cross-compare changes in cytokine concentration, instead of presence or absence alone.

During cellular proliferation, IL-1, IL-2, and IL-3 have primary roles in maintaining the ocular surface and replacing damaged cells, as well as acting as a sort of alarm system that triggers allergic pathways once they reach a certain concentration [23]. Similarly, IL-4 is present at basal levels and interactions between other cytokines and immune cell subtypes can stimulate a rapid upregulation of this cytokine that leads to the activation of immunological responses [23]. IL-8 is upregulated by cytokines, such as IL-1, in the event of ocular surface invasion, and is used to chemotactically attract relevant leukocytes to the area [23]. IL-10 then reduces cytokine expression on local immune cells by inhibiting allergic response pathways. IL-1, TNF, and IL-6 are all linked in the centre of these immunological responses and impact the upregulation of one another in response to various changes in the ocular surface environment [23]. Cumulatively, most cytokines on the ocular surface work in conjunction to ensure a stable, lubricated, and healthy homeostatic environment.

### 6.2. Cytokines as Biomarkers

The current research in ocular allergy focuses on using cytokines as biomarkers by quantifying their concentrations to diagnose immune and genetic disorders [24,43,44]. The current studies have identified a number of key cytokines as potential biomarkers of ocular allergy using tear samples for cytokine detection assays, such as multiplex, immunoblotting, or enzyme-linked immunosorbent assays (ELISA). Studies conducted in the last few years alone have used biosignatures as diagnostic measures in neuroinflammatory disorders [45], tuberculosis [46], and even paediatric asthma [47]. The pluripotent nature of cytokines, as highlighted in Table 3 above, makes it difficult to directly attribute the presence or absence to a disease state, and may thus be more effective to measure concentrations and develop a baseline volume of particular cytokines as biomarkers [48]. Previously defined biosignatures for ocular allergy are shown in Table 4 below, where the same cytokines are flagged consistently in multiple conditions of the eye. As well as this high crossover of function, cytokine levels vary from person to person reliant on diet, immune state, age, stress, and health [49]. Countermeasures for this include high participant numbers to ensure even representation or classifying signatures by subgroup (i.e., one unique biosignature per age group). Assays that detect combinations of cytokines in a sample could generate unique reference biosignatures for that disease as a new diagnostic tool [50].

Most cytokines shown in Table 4 play a role in inflammatory pathways in ocular conjunctiva either as a response to allergens or pathogens [57]. A recent collection of papers by Ghasemi et al. looked at single-cytokine and ocular surface interactions in contact lens wearers [58]. Data collected on tear cytokine levels between studies appear highly varied and inconsistent [58]. It is not feasible to draw comparisons between studies such as those included in the review articles outlined in Table 4 above, due to high variability in participant age, genetics, sex, location, sample collection method, and assay types. Alternative collections methods such as sponge absorptions, microcapillary tube suction, or Schirmer strip absorption can yield a varying degree of results as Schirmer strips and sponges both cling to cytokines, making their removal from the strips difficult and, thus, reducing the capacity for analysis [59]. Assay types are most commonly ELISA, multiplex magnetic bead analysis or flow cytometry [60,61]. Each set of variables is applied to generally small (*N* < 100) datasets, leading to high variations in cytokine biosignatures [62]. Conducting a series of uniform studies amongst a number of groups would generate a much more thorough dataset with high potential for generalisation to a wider population, such as keratoconus sufferers and those at risk of developing it. Thus, it is important to ensure that the detection methods for these cytokines are sufficiently sensitive and cost-effective for the potential development of clinical applications.

A study by Willcox et al. from 2015 states that degree of ocular irritation is not directly related to cytokine concentration variations [63]. However, the validity of this assessment is called into question as measures of irritation were subject to participant bias [64]. For example, subjects were asked to grade themselves on a scale from 1 to 100, but since irritation is subjective, the results may not be 100% representative of the truth [63]. This raises an important point regarding the development of an unbiased measurement of irritation. However, it is also known that the most consistent evidence for allergy is the symptom of allergy and, thus, this is used as the hallmark for diagnosis. The unfortunate downside of this is an inability to detect specific environmental triggers and, this way, one can only estimate links between certain behaviours, like diet or reactions to topical creams, for example. Unbiased measures may include assessment by optometrists to ensure participants are consistently evaluated using validated grading scales for ocular inflammation, redness, papillae, and by correlation to cytokine concentrations [65]. Utilization of hypersensitive detection methods for cytokines with minimal opportunities for human error and high repeatability is, therefore, imperative to ensure future studies are robust and not subject to bias [35].

Future prospects for this field are plentiful. Full cytokine biosignature characterisation for ocular allergy will allow research to progress to flow cytometry as a means of investigating intracellular cytokine roles in the allergic pathway on the surface of the eye [66]. Linking physiology and cellular biomarkers may kickstart new optometry and allergy crossover studies. Consolidating foundations of broad papers by conducting murine ocular model studies that test hypotheses regarding cytokine function would be a beneficial continuation [20]. Additionally, school-wide allergy questionnaires and visual acuity tests to gain prevalence data among youth populations would be novel and contribute to raising awareness for the importance of ocular health during ocular allergy flare-ups.

Improvement of current treatment options is a potentially important future field of research. Typical treatment options for ocular allergy include anti-histamines and mast cell stabilizers, taken orally either before or during a reaction to reduce degranulation of immune cells [18]. Intranasal corticosteroids and antihistamine sprays are also effective at reducing ocular symptoms by inhibiting the action of inflammatory mediators, though work most effectively as a preventative measure [67]. As a result, subcutaneous immunotherapy (SCIT) is considered the most effective preventative method of treatment as it targets the cause of allergy as opposed to the response to exposure [68]. The benefits of monthly SCIT treatments have been proven to last up to 2 or 3 years after the last treatment session [68]. Each round of subcutaneous injection contains a small volume of the allergen with the intention of gradually increasing dosage over time [69]. In doing so, physicians effectively desensitize the body to the allergen and reduce the severity and occurrence of allergy [69]. The unfortunate downside to this method of treatment, however, is its costly and highly invasive nature, as well as the fact that it is not tailored for ocular allergy. The development of non-invasive, highly effective, and inexpensive alternatives is a very lucrative field of research.

Utilising cytokine-centric ocular allergy research, development of a drug that directly inhibits cytokine signalling on the ocular surface, in the form of eye-drops or oral tablets, could be a universally beneficial alternative that is not subject to individual sensitivities. Similar technologies are currently being developed, with a study by Chassin et al. in 2017 reporting the development of a lab-synthesised dual Th2 cytokine sensor (DCS) device modelled on mammalian cells [70]. This DCS device is potentially capable of detecting interaction levels between IL-4 and IL-13, and subsequently triggering the release of designed ankyrin repeat protein (DARPin) E2_79 when an allergic reaction is recognised [70]. DARPin E2_79 is then able to bind to IgE and thus dampen the degranulation of local immune cells that leads to physiological symptoms [70]. Alternatives to this method of inactivating the allergic pathway include anticytokine drugs that can be orally administered to prevent allergic flare-ups.

Drugs like tralokinumab are currently being used in treatment of allergic disorders such as atopic dermatitis [71]. Tralokinumab works in preventing allergy by targeting the IL-13 receptors on effector cells, though there is no research to support the application of this particular drug in ocular allergy [71]. In fact, there are no currently available ocular allergy treatments that rely on anticytokine mechanisms of action. This provides a very interesting future avenue for research that could lead to the development of preventative eye-drops or oral tablets that greatly reduce the symptoms of ocular allergy.

A paper by Bahn in 1998 suggests that the lack of current anticytokine therapies is due to the unpredictable and potentially devastating impact of inhibiting singular cytokine production as a result of their highly pluripotent nature [72]. As such, the direct impacts are difficult to quantify and may vary highly between individuals [72]. Additionally, the degree of reduction or increase in production of cytokines will vary drastically between individuals due to regulatory levels within localized cells [73]. Each person will have different concentrations, different sensitivities, and different cellular-level reactions to any number of pathogens, allergens, foreign bodies, injury, or even their own cells, based on factors like age, sex, genetics, and disease states [73]. It is for this reason that cytokine biosignatures are also not necessarily a gold standard characterisation of individual hypersensitivity and may only be used as a guideline to gauge both the degree of reaction and generalized profiles that can lead to more progressive and specific research in this field that utilizes proteomics and more sensitive quantification methods. Instead, Bahn suggested using localized gene therapy to alter cytokine secretion and prevent over- or underexpression at the source, thus reducing the effects of individual variances in basal cytokine secretion that acts as a major limiting factor for most anticytokine therapeutics [72].

In addition, this proposed meeting of optometry and biomedical science could spark the future development of hypoallergenic contact lenses to reduce the impact of allergens on the eye while functioning regularly to correct vision. Such a product would be revolutionary in optometry, and could drastically alter the impact of eye rubbing-induced vision loss and corneal degradation that is currently on the rise. It would also contribute to the not-yet consolidated, although thoroughly hypothesised link between ocular allergy, contact lens wear, and corneal degradation that may progress further to corneal ectasia [74]. More research into these links would be highly beneficial for contact lens-wearing subgroups suffering from ocular allergy and who wish to know more about the cumulative effect of cytokine interactions on the eye, and whether or not they contribute to their risk for ocular disease.

## 7. Conclusions

Laying the groundwork for cytokine biosignature studies in ocular allergy is imperative for ensuring bigger-picture disease research is based on factual and robust data. Compiling an ocular allergy biosignature does not only impact this subsection of the global population but provides a framework for investigating cytokine pathways in ocular degeneration which could change the future of keratoconus sufferers by shedding light on the cellular basis of gradual thinning and sagging of the cornea they experience, and may contribute to prevention or treatment [75]. Beginning with characterisation of ocular allergy biosignatures could improve diagnosis and may spur development of new testing methods for identification of allergic triggers that are non-invasive, cheap, and effective. The applications are endless, and the future of ocular health research is bright.

## Figures and Tables

**Table 1:** Summary of the key similarities and differences in both pathophysiology and symptomatic physiology between those suffering from seasonal allergic conjunctivitis (SAC), perennial allergic conjunctivitis (PAC), vernal keratoconjunctivitis (VKC), or atopic keratoconjunctivitis (AKC) [18,19].

	SAC	PAC	VKC	AKC
Presentation	Intermittent	Persistent	Intermittent and persistent phases	Chronic
Allergic mechanism	IgE-mediated	IgE-mediated	IgE and non-IgE mediated	IgE and non-IgE mediated
Cell types involved	Mast cells	Mast cells	Mast cells, eosinophils, lymphocytes, basophils, plasma cells, and macrophages	Mast cells, eosinophils, and lymphocytes
Background	Atopic	Atopic	Childhood and/or atopic	Atopic
Eyelids	Oedema	Oedema	Oedema, pseudoptosis (saggy eyelid)	Eczema, meibomitis (tear gland inflammation), blepharitis (oil gland inflammation)
Conjunctiva	Follicles and/or papillae	Follicles and/or papillae	Giant papillae	Papillae and/or fibrosis
Limbus	No effect	No effect	Thickening, Trantas dots	Thickening, Trantas dots
Cornea	No effect	No effect	Ulcer, vernal plaques	Ulcer, vernal plaque, opacities, neovascularization
Discharge	Clear mucoid	Clear mucoid	Stringy mucoid	Stringy mucoid
Symptoms	Watery eyes, itchy eyes, inflammation, discomfort, conjunctivitis, swollen eyelids, blurred vision	Watery eyes, itchy eyes, inflammation, discomfort, conjunctivitis, swollen eyelids, blurred vision	Itchy eyes, irritation, discomfort, conjunctivitis, photosensitivity, papillae	Itchy eyes, irritation, discomfort, conjunctivitis, swollen eyelids, papillae, photosensitivity

**Table 2:** Summary of the actions of cytokines detected on the ocular surface in ocular allergy and contact lens wear. Adapted from Broide et al. to specify allergy and cytokine interactions [21,30,31,32,33]. IL, interleukin; TNF, tumour necrosis factor; IFN, interferon.

Cytokine	Actions
IL-1β	Induces fever Proinflammatory Stimulates synthesis of other cytokines
IL-2	Promotes allergic reactions
IL-4	Upregulates allergic reaction mediator cells
IL-5	Increases proliferation of allergic B cells in eosinophil-mediated ocular allergy
IL-6	Promotes allergic cell differentiation
IL-8	Chemotactic
IL-10	Inhibition of allergic responses Downregulation of inflammatory cytokines
IL-12	Supresses allergy Upregulates IFNγ
IL-13	Promotes antibody production Upregulates chemotactic cytokines Inhibits cytokine secretion
TNFα	Chemotactic Increases cytokine secretion
IFNγ	Inhibits allergic reactions

**Table 3:** Regulatory and inflammatory cytokines present in varying concentrations on the ocular surface. Adapted from [23]. IL, interleukin; TNF, tumour necrosis factor.

Cytokine	Role within the Eye
IL-1	Triggers production of IL-2, IL-6, IL-8, and TNFα Stimulates cellular proliferation
IL-2	T cell activation and proliferation begins when this cytokine reaches the requisite concentration
IL-3	Growth and proliferation of pluripotent stem cells to replace old or damaged cells
IL-4	Starts off the Th2 allergic response
IL-6	Cellular growth Production of antibodies
IL-8	Chemotaxis—chemical gradient attraction of cells from one location to another
IL-10	Inhibits cytokine synthesis to stop a cellular reaction
TNFα	Activates T-cells Stimulates IL-1 and IL-6 synthesis

**Table 4:** Overview of recent literature and measured cytokine concentrations in seasonal allergic conjunctivitis (SAC), perennial allergic conjunctivitis (PAC), vernal keratoconjunctivitis (VKC), atopic keratoconjunctivitis (AKC) from 2003 to 2019 [11,16,23,40,43,44,51,52,53,54,55,56,57]. IL, interleukin; TNF, tumour necrosis factor; IFN, interferon.

Source	SAC	PAC	VKC	AKC
Leonardi, Motterle and Bortolotti, 2008 [11]	-	-	IL-8, IFNγ, IL-4, IL-13	-
Leonardi, 2013 [16]	IL-4, IL-5, IL-13, IL-1β	-	IL-4, IL-5, IL-10, IL-12, IL-13, IFNγ, IL-1β, IL-6, TNFα	IL-2, IL-4, IL-5, IL-10, IFNγ, IL-1β, IL-6, TNFα, IL-8
Bonini et al., 2003 [23]	IL-2, IL-4, IL-5, IL-10, TNFα, IFNγ	IL-2, IL-4, IL-5, IL-10, IFNγ	IL-1β, IL-6	-
Leonardi et al., 2006 [51]	IL-1β, IL-2, IL-4, IL-5, IL-6, IL-10, IL-12, IL-13, IFNγ,	-	IL-1β, IL-2, IL-4, IL-5, IL-6, IL-10, IL-12, IL-13, IFNγ, TNFα	IL-1β, IL-2, IL-5, IL-6, IL-12, IL-13
Cook, 2004 [52]	IL-1β	-	IL-4, IL-5, IL-10, IL-12, IL-13, IFNγ, IL-1β, IL-6, TNFα	IL-2, IL-4, IL-5, IL-10, IFNγ, IL-1β, IL-6, TNFα,IL-8
Enriquez-de-Salamanca and Calonge, 2008 [54]	IL-1β, IL-2, IL-4, IL-5, IL-6, IL-12, IL-13, IFNγ	-	IL-1β, IL-2, IL-4, IL-5, IL-6, IL-12, IL-13, IFNγ, TNFα	IL-1β, IL-2, IL-4, IL-5, IL-6, IL-10, IL- 12, IL-13, IFNγ, TNFα
Di Zazzo et al., 2017 [56]	-	-	IL-4, IL-5, TGF-β1, IL-1β, IL-6, TNFα	IL-4, IL-5
